# Scene and human face recognition in the central vision of patients with glaucoma

**DOI:** 10.1371/journal.pone.0193465

**Published:** 2018-02-26

**Authors:** Alexia Roux-Sibilon, Floriane Rutgé, Florent Aptel, Arnaud Attye, Nathalie Guyader, Muriel Boucart, Christophe Chiquet, Carole Peyrin

**Affiliations:** 1 Univ. Grenoble Alpes, CNRS, LPNC, Grenoble, France; 2 Department of Ophthalmology, University Hospital, Grenoble, France; 3 Department of Neuroradiology and MRI, University Hospital, Grenoble, France; 4 Université Grenoble Alpes, CNRS, GIPSA-Lab UMR 5210, Grenoble, France; 5 Université de Lille, CNRS, SCALab UMR 9193, Lille, France; Macquarie University, AUSTRALIA

## Abstract

Primary open-angle glaucoma (POAG) firstly mainly affects peripheral vision. Current behavioral studies support the idea that visual defects of patients with POAG extend into parts of the central visual field classified as normal by static automated perimetry analysis. This is particularly true for visual tasks involving processes of a higher level than mere detection. The purpose of this study was to assess visual abilities of POAG patients in central vision. Patients were assigned to two groups following a visual field examination (Humphrey 24–2 SITA-Standard test). Patients with both peripheral and central defects and patients with peripheral but no central defect, as well as age-matched controls, participated in the experiment. All participants had to perform two visual tasks where low-contrast stimuli were presented in the central 6° of the visual field. A categorization task of scene images and human face images assessed high-level visual recognition abilities. In contrast, a detection task using the same stimuli assessed low-level visual function. The difference in performance between detection and categorization revealed the cost of high-level visual processing. Compared to controls, patients with a central visual defect showed a deficit in both detection and categorization of all low-contrast images. This is consistent with the abnormal retinal sensitivity as assessed by perimetry. However, the deficit was greater for categorization than detection. Patients without a central defect showed similar performances to the controls concerning the detection and categorization of faces. However, while the detection of scene images was well-maintained, these patients showed a deficit in their categorization. This suggests that the simple loss of peripheral vision could be detrimental to scene recognition, even when the information is displayed in central vision. This study revealed subtle defects in the central visual field of POAG patients that cannot be predicted by static automated perimetry assessment using Humphrey 24–2 SITA-Standard test.

## Introduction

Primary open-angle glaucoma (POAG) is an ocular disease characterized by a progressive destruction of retinal ganglion cells and the optic nerve. This results in progressive vision loss affecting mainly peripheral vision [1]. However, this visual field loss is rather different than the black tunnel vision that patients are believed to experience. Vision loss includes missing parts and blurred distortions [2,3]. As POAG develops slowly and without pain, the diagnosis is often made at an advanced stage. By this time the visual handicap is irreversible. Therefore, it is important to develop and propose visual tests and tools to diagnose the disease at early stages. Then disabilities can be quantified at different stages, to identify individuals who might benefit from rehabilitative intervention. As the disease is due to the destruction of retinal ganglion cells, studies of POAG usually focus on low-level aspects of visual processing, i.e. retinal sensitivity to visual stimulation. The visual field defect is mostly assessed by static automated perimetry. This is based on luminance increment detection of small dots and allows evaluation of retinal sensitivity. However, POAG patients often report discomfort in their daily life, even at stages when static automated perimetry only indicates a slight peripheral vision loss [4]. Furthermore, the current state of our understanding of visual cognition implies that simple aspects of visual information are integrated into more complex percepts. Retinal sensitivity loss may thus impact more complex aspects of visual processing in POAG patients, even early in the disease. Converging descriptions of complex visual deficits in glaucoma include form and motion extraction [5], face and object recognition [6,7], reaching and grasping [8], mobility [4], executions of natural actions [9]. Also, specific eye movement patterns have been observed during reading [10], face recognition [11], exploration of scenes [12,13,14], visual search of objects [15].

Importantly, there is also evidence of complex visual impairment, even in areas of the visual field classified as normal by static automated perimetry [5,7]. Usually POAG causes a decrease in contrast sensitivity, correlated with the visual field defect [16]. However, Lenoble et al. [7] showed that people diagnosed with glaucoma, without central visual field loss as reflected by static automated perimetry, performed worse than controls in categorizing single objects presented foveally at diminished luminance contrast of stimuli. These results suggest that recognition abilities of patients may be more impacted than mere retinal sensitivity (i.e. detection of small dots).

The aim of the present study was to assess the visual abilities of POAG patients with respect to the perception of scene photographs (Scene experiment) and human face photographs (Face experiment) in the central visual field, a region considered to be relatively preserved and only affected in the later stages of the disease. The critical manipulation in these experiments was the level of visual processes required to perform a task, while presenting the same visual stimuli. For this purpose, we first evaluated patients' retinal sensitivity using a detection task of photographs of scenes or human faces, with instructions mimicking those of static automated perimetry. We then evaluated the recognition of these photographs with a categorization task. Primordially, we tested the participants' ability to detect and categorize the same stimuli, which enabled us to directly compare performances in detection (low-level visual processes) and categorization (higher-level visual processes). We considered the difference in performance between detection and categorization to be a reliable indicator of the cost of higher-level visual processes. POAG patients were divided into two groups: patients with a partially affected central visual field, and those with a preserved central visual field, as observed by perimetry using Humphrey 24–2 SITA-Standard test. Performances of the two groups of patients were compared to age-matched normally sighted participants. According to static automated perimetry results, we expected that only patients with a central defect (CD) would have altered capacities for low-level processes (the detection task), with respect to controls. Further, we proposed that the peripheral field loss would impact high-level functions in central vision in a way not predicted by the Humphrey 24–2 SITA-Standard test. We expected a categorization deficit, with respect to controls, not only for patients with a central visual field defect but also for those with no central defect (NoCD). Additionally, we considered the impact of luminance contrast of the stimuli. Two low levels were presented (10% and 2.5%), reproducing the situation of twilight or a darkened room. According to the results of Lenoble et al. [**7**], we expected that the difference of performance between the Detection and the Categorization tasks would be larger for the lowest contrast level (2.5%), and that this interaction would be stronger for NoCD patients than for controls.

## Methods

### Participants

**Fig 1:**
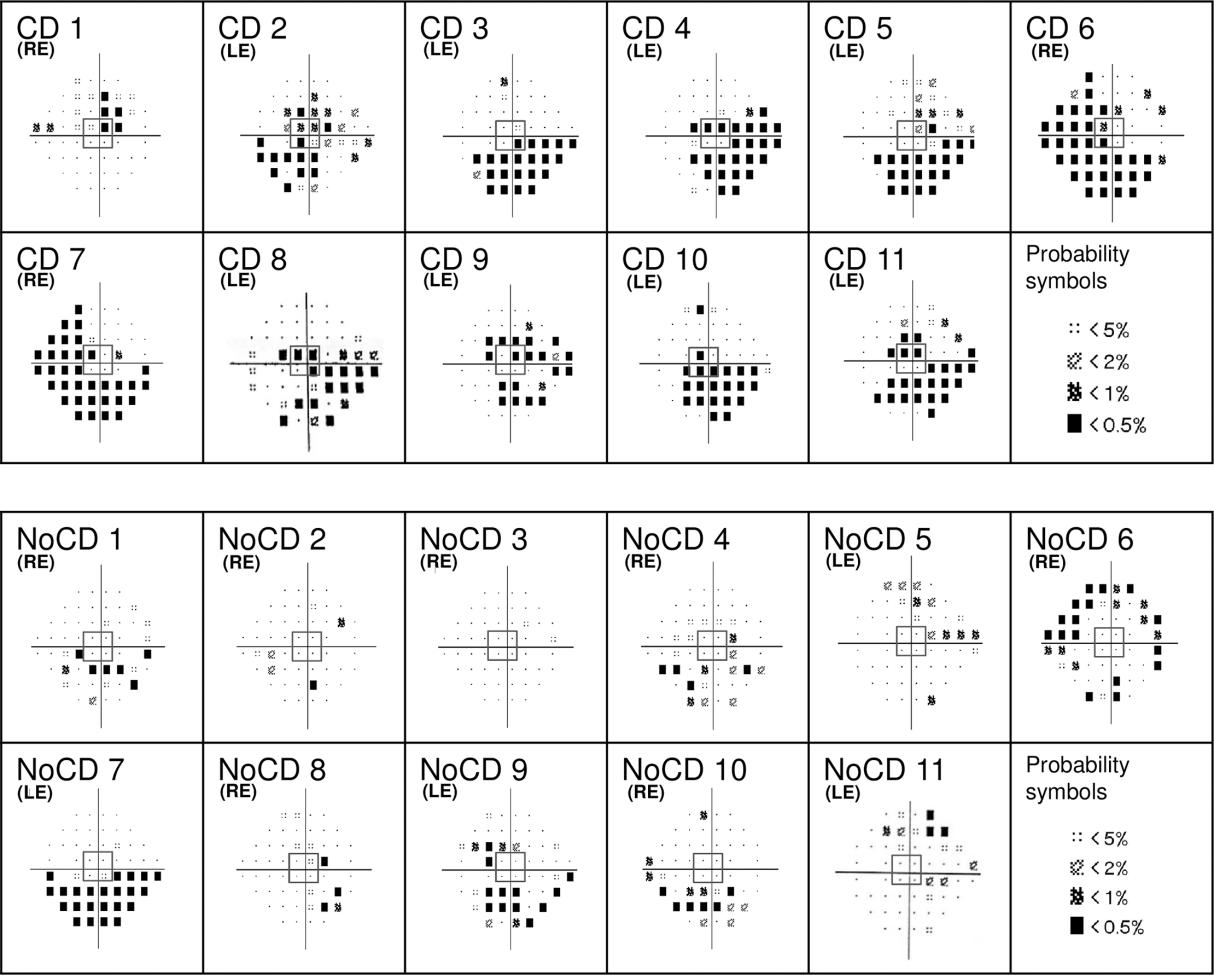
Individual mean deviation probability plots for patients with primary open-angle glaucoma. A deficit in the central visual field was defined by at least one of the four central points (framed in central grey squares) presenting a probability of less than 2% of being normal. The tested eye is indicated for each patient (LE: left eye; RE: right eye). CD: Patients with central defect; NoCD: Patients with no central defect.

Twenty-two subjects (12 women) currently being treated with topical therapy for POAG were included in the experiment. Diagnosis was established in the ophthalmologic department of the Grenoble Alpes University Hospital. Patients participated in this non-interventional behavioral experiment following their clinical examination. The best corrected visual acuity in the eye to be tested was at least 6.3/10 on a Monoyer scale for distance vision (or 0.2 logMAR) and about 1.5 or 2 Parinaud for near vision. If both eyes corresponded to these criteria, the tested eye was chosen to balance the two POAG groups (with or without a central visual field defect). Monocular assessment of POAG patients’ visual field was performed with adapted corrected lenses, through a Humphrey Visual Field Analyzer (Carl Zeiss, Meditec, Dublin, CA; 24–2 SITA-Standard procedure) in each eye. The quality of the patients’ central visual field was examined based on the individual deviation probability plot of the four central points tested, which cover the central 6° of the visual field (3° in each hemifield). A deficit in the central visual field was defined by at least one of the four points presenting a probability inferior of 2% of being normal. This classification resulted in two POAG groups (Fig 1); 11 patients with a CD (5 women), and 11 NoCD patients (7 women). Mean visual acuity for CD patients was 9.6/10 (± 0.9) Monoyer, and 2 Parinaud for all patients. Mean visual acuity for NoCD patients was 9.5/10 (± 1.0) Monoyer, and 2 Parinaud for all patients. Twenty-five control subjects (14 women) were also tested unilaterally, in the eye with the best corrected visual acuity (Control group). Mean visual acuity for the Control group was 10/10 Monoyer and 2 Parinaud for all participants. Control subjects also underwent monocular automatized Humphrey visual field assessment with a 24–2 SITA-Standard procedure in each eye to ensure the absence of campimetric deficit.

The three groups were approximately age-matched (Control group: 68.0 ± 6 years, range 54–83 years; CD group: 67.9 ± 10 years, range 52–79 years; NoCD group: 67.1 ± 9 years, range 55–79 years; *t-tests*: Control vs. CD: *t(34)* = 0.03, *p* = 0.97; Control vs. NoCD: *t(34)* = 0.31, *p* = 0.76; CD vs. NoCD: *t(20)* = 0.18, *p* = 0.86). A complete ophthalmological examination (best corrected visual acuity, Goldmann tonometry, anterior segment, and fundus examination) was performed for all participants. None exhibited cognitive impairments (mini mental state examination score > 24/30). Participants with psychiatric, neurological, and ocular disorders (age-related macular degeneration, cataract except for uncomplicated cataract surgery) were not included in the study, and all participants gave their informed written consent before participation. The study was carried out in agreement with the Code of Ethics of the World Medical Association (Declaration of Helsinki) and approved by the local ethics committee (CERNI, COMUE Université Grenoble Alpes, IRB00010290).

### Stimuli and procedure

**Fig 2:**
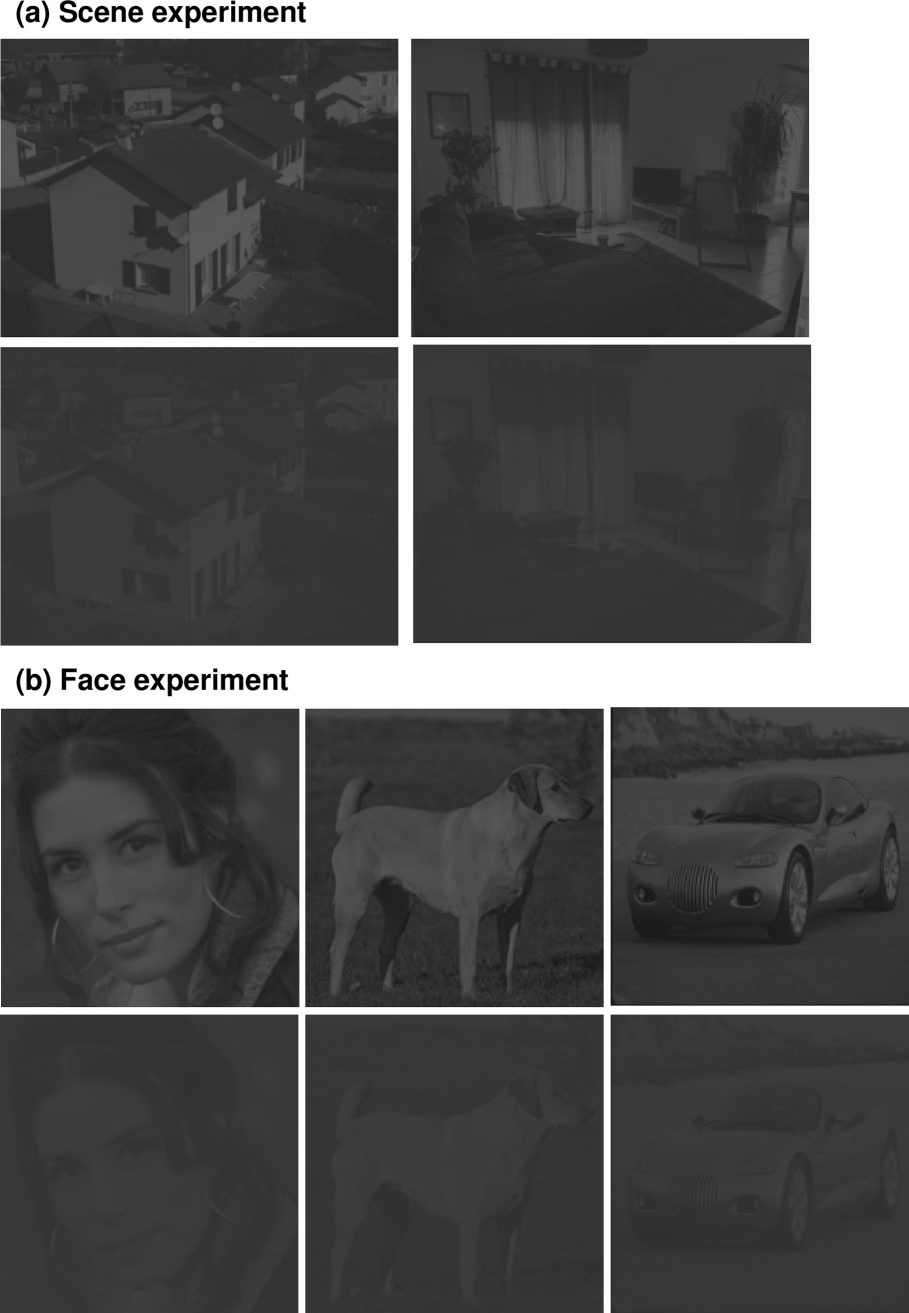
**Examples of stimuli for (a) Scene and (b) Face experiments.** Images were presented at two contrast levels (10% and 2.5%). For illustrative purposes, the contrast level of the images was slightly increased.

All participants performed two successive experiments, a Scene experiment and a Face experiment (Fig 2). Stimuli were elaborated using the MATLAB image processing toolbox (Mathworks Inc., Sherborn, MA, USA). Stimuli in the Scene Experiment were selected from previous research [17,18]. These were gray-scale photographs of 20 scenes (192 × 144 pixels) belonging to two semantic categories: indoors (kitchens, living-rooms) and outdoors (streets, buildings, houses). Both categories were equivalent in terms of visual cluttering and all scenes had a similar energy distribution in both spatial frequency and dominant orientations (similar mean amplitude spectrum). Thus, categorization based on physical properties of the images were avoided. Stimuli in the Face experiment, selected from previous research [19], were gray-scale photographs of 10 human faces, five animals, and five vehicles (192 × 192 pixels). Stimuli were displayed using E-prime software (E-prime Psychology Software Tools Inc., Pittsburgh, USA) on a 30’ monitor, with a resolution of 2560 × 1600 pixels, at a refreshing rate of 60 Hz and with a viewing distance of 38 cm. Optical correction was used for participants requiring visual correction at this distance. Stimuli covered the central 6° of the visual angle width at this viewing distance. Hence, the region covered by the four central points of the automatized visual field evaluation used to determine whether POAG patients presented a central defect was matched. To maintain the distance and central position, participants’ heads were supported by a chinrest. We measured the gamma function of our monitor (i.e. the luminance of the display for different values of uniform gray level stimuli generated with Matlab) using a calibration tool (Spyder5Elite). We then applied a gamma-correction to each stimulus to linearize the gamma function. Decreasing luminance contrast of the images had a proportional effect on the low (dark shades) and high (bright shades) luminance values. We then created two contrast level versions of each image, by manipulating the Root-mean-square contrast (RMS; standard deviation of the luminance [20]). This manipulation resulted in one version of the image with a luminance contrast level of 2.5% and one version with a luminance contrast level of 10% (RMS contrast of respectively 0.007 and 0.03 for luminance values between 0 and 1; see Fig 2). The mean luminance values of all images as well as the background luminance of the screen were set at 72 on a 256 gray-level scale (0.28 for luminance values between 0 and 1; 10 cd/m^2^) to match the luminance of the Humphrey Visual Field Analyzer background (luminance of 31.5 apostilbs, corresponding to approximately 72 gray-levels or 10 cd/m^2^).

**Fig 3:**
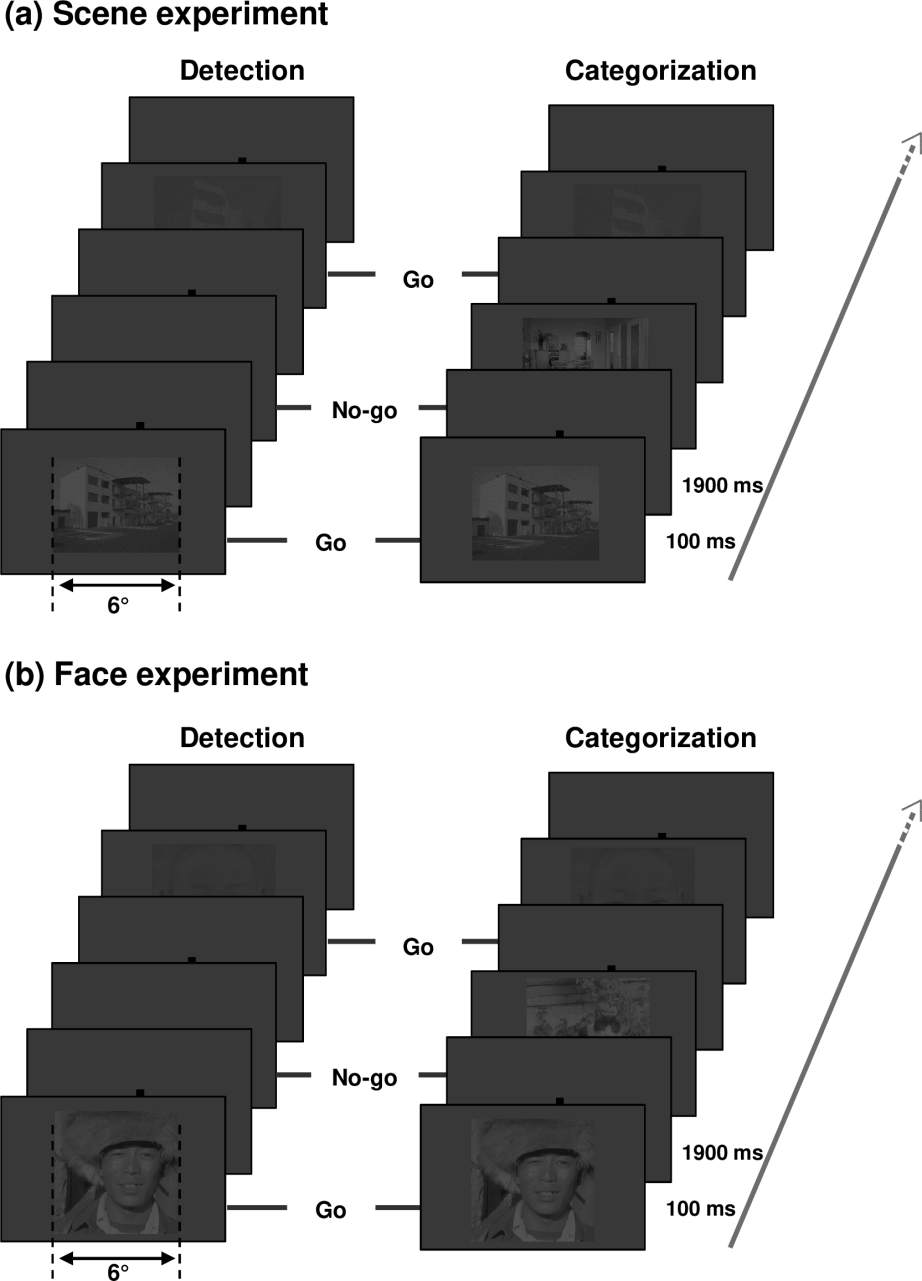
**Schematic procedure for (a) Scene and (b) Face experiments.** For each experiment, examples of two *go* trials and two *no-go* trials are represented by task (Detection and Categorization). Visual stimulation for *go* trials was the same for the two tasks. Images were presented in two contrast levels (10% and 2.5%). For illustrative purposes, the contrast level of the images was slightly increased.

Each experiment (Scene and Face) consisted of two experimental blocks dedicated to two different tasks: a Detection task and a Categorization task (Fig 3). For both experiments, the Detection task was to press the keyboard spacebar when a stimulus (a scene or a human face) appeared on the screen, based on a go/no-go trial paradigm. The Detection task consisted of 20 *go* trials and 20 *no-go* trials. In *go* trials, an image (a scene or a face) was presented against the uniform grey background (10 trials for each contrast level). In *no-go* trials, there was no image, only the uniform grey background. Participants were explicitly told not to try to recognize the image. However, they had to respond as quickly as possible, as soon as they could see the presence of an image. For the Categorization tasks, participants had to press the keyboard spacebar when the image belonged to a target category, but not when it belonged to a distractor category. Target and distractor categories for the Scene experiment were counterbalanced across participants (either indoors as target and outdoors as distractors, or the inverse). In the Face experiment, the target category was always faces and distractors were always animals or vehicles. Participants were told to respond as quickly as possible, as soon as they could see an image belonging to the target category. The Categorization task consisted of 20 *go* trials (target category, 10 for each contrast level) and 20 *no-go* trials (distractor categories, 10 for each contrast level). In each experiment, *go* trial stimuli were the same in the detection and categorization tasks; only task demands changed.

A stimulus (either a photograph or nothing for *no-go* trials of the Detection block) was displayed against the uniform grey background for 100 ms to allow a sufficient processing time while minimizing saccades. A central fixation point was displayed against the uniform grey background before and after the trial stimulus (an image against the grey background or only the grey background) to control the gaze direction to the center of the screen. The inter-stimulus interval was 1900 ms. Scene and Face experiments were counterbalanced across participants of each group. In each experimental session, the Detection task always preceded the Categorization task, to avoid contamination of the Detection task by categorization processes. Contrast conditions in each block were presented in random order. Before each experimental block, participants underwent a short training block comprising 12 trials using stimuli that differed from the ones used in the experiment. The participant's response was recorded for each trial (either a *go*-response or a *no-go*-response) and reaction times were recorded for the *go* responses.

### Data analysis

The participants’ responses were designed for each trial as follows: correct detection of a target (an image in the Detection task or a specific category in the Categorization task) was a hit, while the absence of a response when a target was present was an omission. Furthermore, a detection of a target when no target was presented was a false alarm and no response when no target was presented was a correct rejection. Based on the proportion of hits, omissions, false alarms, and correct rejections, a d’ index of sensitivity was calculated for each participant and each experimental condition, as part of the Signal detection theory. One analysis of variance for the Scene experiment and one for the Face experiment were then carried out on the d’ index of sensitivity using Statistica 10.0 software (Statsoft, Tulsa, USA). The between-subject factors were Group (Control, CD, and NoCD), and within-subject factors were Task (Detection and Categorization) and Luminance contrast level (2.5% and 10%). Effect sizes were estimated by partial eta-squared (η^2^). The significance level of tests was set at 0.05. Due to the presence of extreme proportions of omission for several patients, especially for the Categorization task when the luminance contrast was 2.5%, the mean correct reaction times could not be calculated. Thus, no analysis was conducted on reaction times.

## Results

### Scene experiment

**Fig 4:**
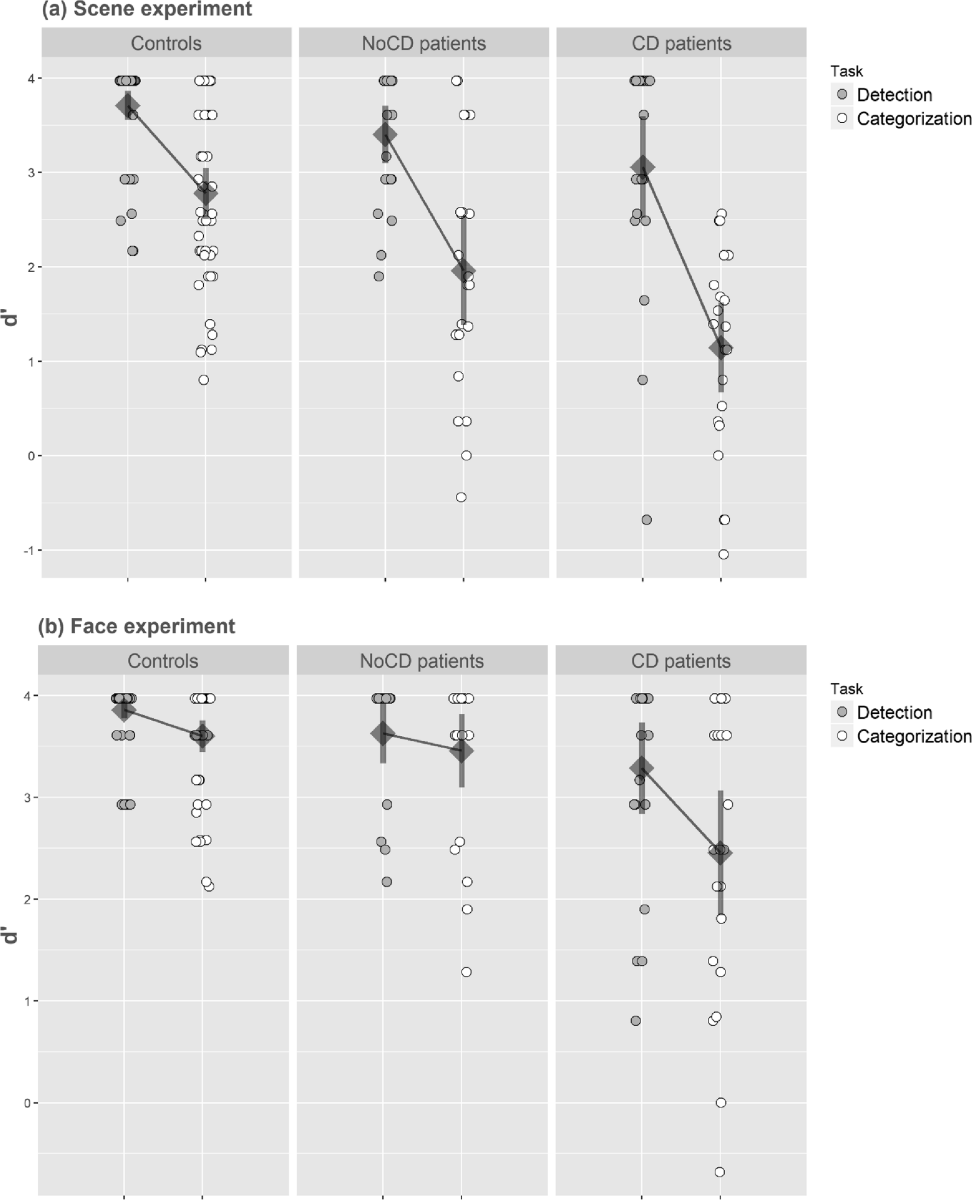
**Index of sensitivity (d’) in (a) the Scene experiment and (b) the Face experiment.** Results are presented as a function of the group (primary open-angle glaucoma patients with central defect: CD; primary open-angle glaucoma patients without central defect: NoCD; and normally sighted age-matched participants: controls), and the task (detection vs. categorization). Error bars correspond to 95% confidence intervals.

The analysis of variance performed on d’ first revealed a main effect of group (F(2,44) = 15.40, *p* < 0.001, η^2^ = 0.41) with a higher sensitivity for controls than for CD patients (controls: 3.24 ± 0.44; CD: 2.68 ± 0.68; F(1,44) = 33.52, *p* < 0.001) and for NoCD patients (controls: 3.24 ± 0.44; NoCD: 2.10 ± 0.74; F(1,44) = 8.82, *p* < 0.05). The main effect of the task was significant (F(2,44) = 176.34, *p* < 0.001, η^2^ = 0.80), with a lower sensitivity for the Categorization task (2.20 ± 1.02) than for the Detection task (3.48 ± 0.60). Furthermore, the Task interacted with the Group factor (Fig 4A; F(2,44) = 8.27, *p* < 0.001, η^2^ = 0.27). Planned comparisons revealed that, for the Detection task, CD patients had a lower sensitivity than controls (CD: 3.06 ± 0.92; controls: 3.71 ± 0.33; F(1,44) = 10.74, *p* < 0.001), while the difference between NoCD patients and controls was not significant (NoCD: 3.40 ± 0.48; controls: 3.71 ± 0.33; F(1,44) = 2.38, *p* = 0.13). For the Categorization task, mean comparisons showed that CD patients, and notably NoCD patients, had a lower sensitivity than controls (CD: 1.14 ± 0.78; NoCD: 1.96 ± 0.98; controls: 2.78 ± 0.67; CD vs. controls: F(1,44) = 33.71, *p* < 0.001; NoCD vs. controls: F(1,44) = 8.45, *p* < 0.01). Considering only controls and CD patients as groups, the Task interacted with the Group suggesting that the difference between sensitivity for the Categorization task and for the Detection task was larger for CD patients than for controls (CD patients: 1.44 ± 0.73; controls: 0.93 ± 0.60; F(1,44) = 15.73, *p* < 0.001). Importantly, considering only controls and NoCD patients as groups, the Task also interacted with the Group suggesting that the difference between sensitivity for detection and categorization was also larger for NoCD patients than for controls (NoCD patients: 1.91 ± 0.82; controls: 0.93 ± 0.60; F(1,44) = 4.26, *p* < 0.05).

**Fig 5:**
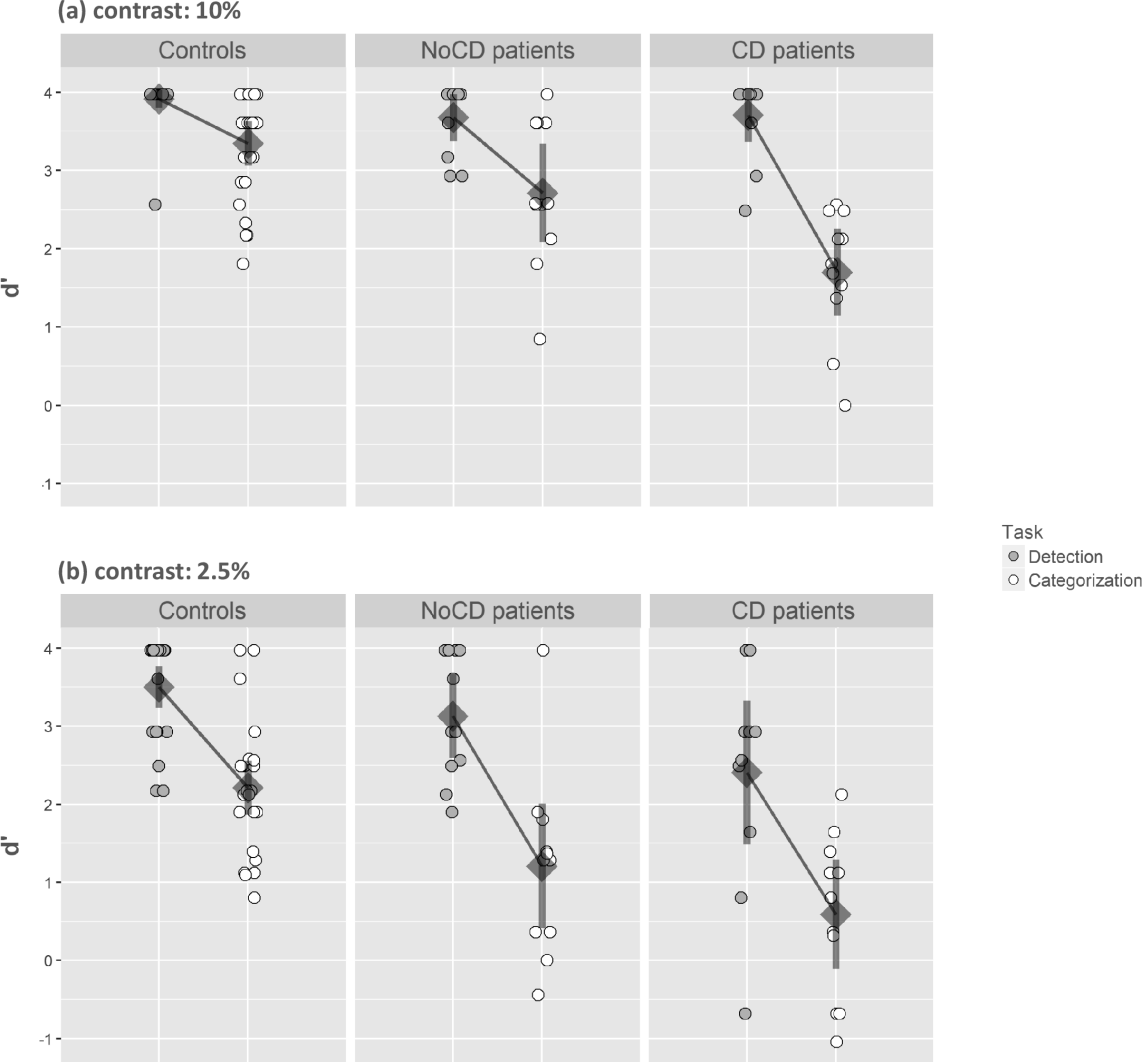
Index of sensitivity (d’) in the Scene experiment. Results are presented as a function of the group (primary open-angle glaucoma patients with central defect: CD; primary open-angle glaucoma patients without central defect: NoCD; and normally sighted age-matched participants: controls), the task (detection vs. categorization), and the contrast level (10% vs. 2.5%). Error bars correspond to 95% confidence intervals.

There was no main effect of the Luminance contrast level (F(2,44) = 1.89, *p* = 0.16) but the interaction between Contrast level × Task × Group was significant (Fig 5; F(2,44) = 3.96, *p* < 0.05, η^2^ = 0.15). When the luminance contrast of scenes was 10%, planned comparisons revealed that, for the Detection task, there was no difference in sensitivity between controls and NoCD patients (controls: 3.91 ± 0.28; NoCD: 3.68 ± 0.45; F(1,44) = 2.94, *p* = 0.09), nor between controls and CD patients (controls: 3.91 ± 0.28; CD: 3.71 ± 0.52; F(1,44) = 2.19, *p* = 0.15). For the Categorization task, mean comparisons showed that CD patients as well as NoCD patients had a lower sensitivity than controls (CD: 1.70 ± 0.82; NoCD: 2.71 ± 0.93; controls: 3.35 ± 0.69; CD vs. controls: F(1,44) = 33.94, *p* < 0.001; NoCD vs. controls: F(1,44) = 5.00, *p* < 0.05). When the luminance contrast of scenes was 2.5%, planned comparisons showed that for the Detection task, CD patients had a lower sensitivity than controls (CD: 2.41 ± 1.37; controls: 3.50 ± 0.44; F(1,44) = 11.55, *p* < 0.05) but there was no difference between controls and NoCD patients (controls: 3.50 ± 0.44; NoCD: 3.13 ± 0.80; F(1,44) = 1.34, *p* = 0.25). For the Categorization task, CD patients as well as NoCD patients had a lower sensitivity than controls (CD: 0.59 ± 1.04; NoCD: 1.21 ± 1.19; controls: 2.21 ± 0.84; CD vs. controls: F(1,44) = 21.28, *p* < 0.001; NoCD vs. controls: F(1,44) = 8.15, *p* < 0.01).

We were interested to see how the level of contrast influenced the difference between detection and categorization performances in each patient group. We first examined the three-way interaction between Group, Task and Contrast, considering only controls and CD patients. This analysis revealed that Contrast had an altered impact on the difference between detection and categorization in these two groups (F(1,44) = 5.83, *p* < 0.05). Indeed, for controls, the difference was larger at 2.5% than at 10% contrast (10% = 0.57 ± 0.73; vs. 2.5% = 1.29 ± 0.85; Task × Contrast interaction: F(1,44) = 11.91, *p* < 0.001), while for CD patients, the difference remained constant between 2.5% and 10% (10% = 2.01 ± 0.92; vs. 2.5% = 1.82 ± 1.00; Task × Contrast interaction: F(1,44) < 1). Then, we examined the three-way interaction, considering only controls and NoCD patients. This interaction was not significant (F(1,44) < 1), suggesting that the contrast level did not have a greater influence on the difference between detection and categorization for NoCD patients (10% = 0.96 ± 0.85; vs. 2.5% = 1.92 ± 0.96) than for controls.

### Face experiment

The analysis of variance performed on d’ first revealed a main effect of the group (F(2,44) = 14.88, p < 0.001, η^2^ = 0.40) with a higher sensitivity for controls than for CD patients (controls: 3.73 ± 0.23; CD: 2.87 ± 0.73; F(1,44) = 28.71, *p* < 0.001). However, NoCD patients performed well overall. There was no difference in sensitivity between controls and NoCD patients (controls: 3.73 ± 0.23; NoCD: 3.54 ± 0.42; F(1,44) = 2.96, *p* = 0.09). The main effect of the Task was significant (F(2,44) = 24.43, *p* < 0.001, η^2^ = 0.36), with a lower sensitivity for the Categorization task (3.30 ± 0.76) than for the Detection task (3.67 ± 0.45). Furthermore, the Task interacted with the Group factor (Fig 4B; F(2,44) = 5.31, *p* < 0.05, η^2^ = 0.19). Planned comparisons revealed that, for the Detection task, CD patients had a lower sensitivity than controls (CD: 3.29 ± 0.65; controls: 3.86 ± 0.19; F(1,44) = 16.24, F(1,44) = 16.24, *p* < 0.001), while the difference between NoCD patients and controls was not significant (NoCD: 3.63 ± 0.41; controls: 3.86 ± 0.19; F(1,44) = 2.63, *p* = 0.11). Similarly, for the Categorization task, mean comparisons showed that CD patients had a lower sensitivity than controls (CD: 2.45 ± 0.95; controls: 3.60 ± 0.44; F(1,44) = 26.80, *p* < 0.001). However, there was no difference between NoCD patients and controls (NoCD: 3.46 ± 0.54; controls: 3.60 ± 0.44; F(1,44) < 1). Considering only controls and CD patients, the Task interacted with the Group (F(1,44) = 8.62, *p* < 0.05) suggesting that the difference between sensitivity for the Categorization task and for the Detection task was larger for CD patients than for controls (CD patients: 0.83 ± 0.70; controls: 0.26 ± 0.49). In contrast, considering only controls and NoCD patients as groups, there was no Group × Task interaction (NoCD patients: 0.17 ± 0.47; controls: 0.26 ± 0.49; F(1,44) < 1).

**Fig 6:**
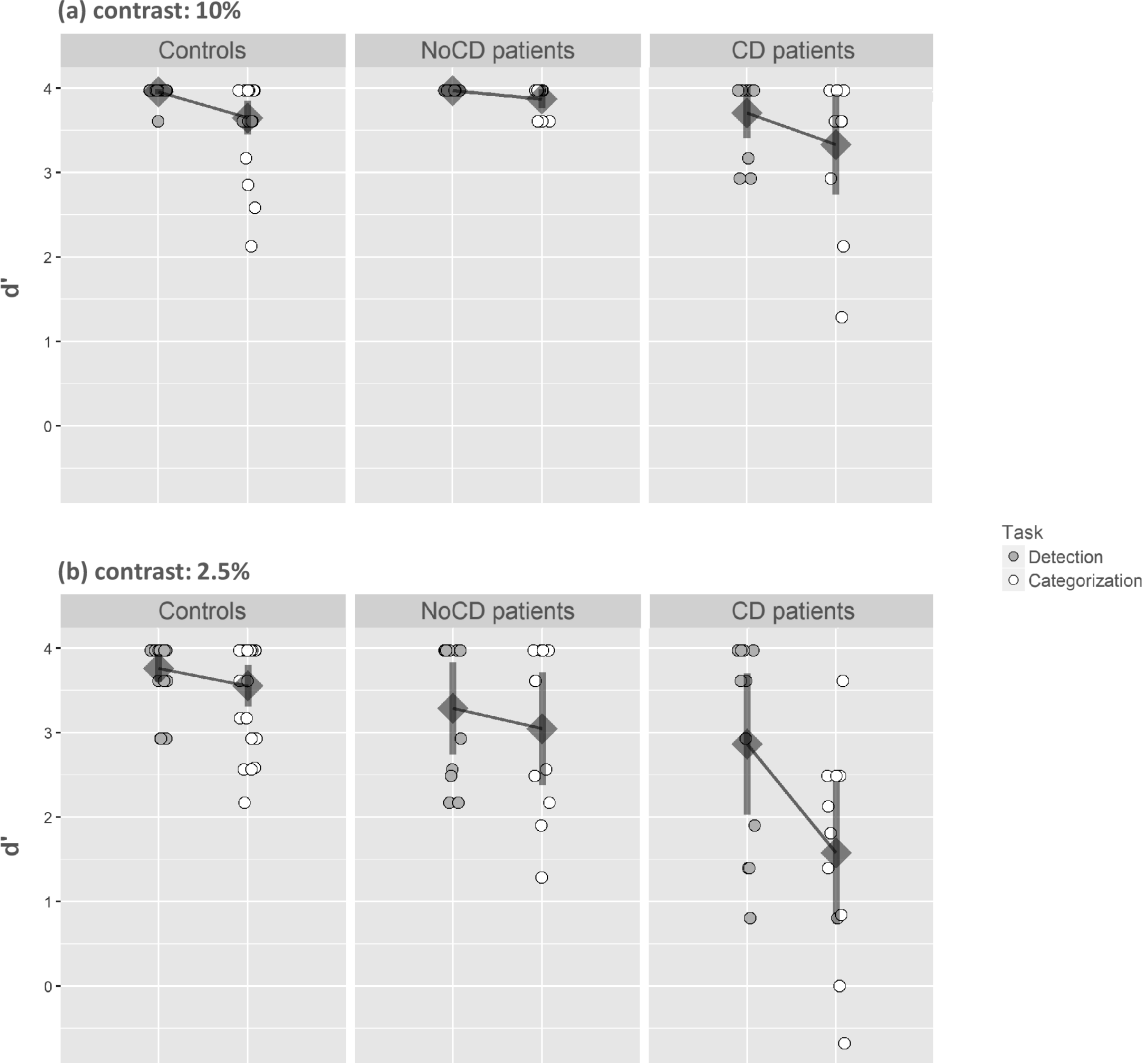
Index of sensitivity (d’) in the Face experiment. Results are presented as a function of the group (primary open-angle glaucoma patients with central defect: CD; primary open-angle glaucoma patients without central defect: NoCD; and normally sighted age-matched participants: controls), the task (detection vs. categorization), and the contrast level (10% vs. 2.5%). Error bars correspond to 95% confidence intervals.

The main effect of Contrast was significant (F(2,44) = 11.83, *p* < 0.001) with a lower sensitivity for 2.5% (3.21 ± 1.01) than 10% (3.76 ± 0.41) and this factor interacted with the Task and the Group (Fig 6; F(2,44) = 4.64, *p* < 0.05, η^2^ = 0.17). When the luminance contrast was 10%, planned comparisons revealed that, for the Detection task, there was no difference in sensitivity between controls and NoCD patients (controls: 3.96 ± 0.07; NoCD: 3.97 ± 0.00; F(1,44) < 1), but controls had a better sensitivity than CD patients (controls: 3.96 ± 0.07; CD: 3.71 ± 0.45; F(1,44) = 9.45, *p* < 0.05). For the Categorization task, there was no difference between controls and NoCD patients (controls: 3.65 ± 0.49; NoCD: 3.87 ± 0.17; F(1,44) = 1.22, *p* = 0.27), or between controls and CD patients (controls: 3.65 ± 0.49; CD: 3.33 ± 0.88; F(1,44) = 2.45, *p* = 0.12). Again, when the luminance contrast was 2.5%, planned comparisons showed that for the Detection task, CD patients had a lower sensitivity than controls (CD: 2.86 ± 1.25; controls: 3.76 ± 0.39; F(1,44) = 10.46, *p* < 0.05) but there was no difference between controls and NoCD patients (NoCD: 3.29 ± 0.81; controls: 3.76 ± 0.39; F(1,44) = 2.94, *p* = 0.09). For the Categorization task, CD patients had a lower sensitivity than controls (CD: 1.58 ± 1.25; controls: 3.55 ± 0.60; F(1,44) = 38.56, *p* < 0.001). In contrast, there was no difference between NoCD patients and controls (NoCD: 3.05 ± 0.99; controls: 3.55 ± 0.60; F(1,44) = 2.55, *p* = 0.12).

We were interested to see how the level of contrast influenced the difference between detection and categorization performances in each patient group, in comparison to controls. We first examined the three-way interaction between Group, Task and Contrast considering only controls and CD patients. This analysis revealed that Contrast had a different impact on the categorization effect in these two groups (F(1,44) = 9.24, *p* < 0.05). For controls, the difference between detection and categorization did not change between 2.5% and 10% contrast (10% = 0.31± 0.50; vs. 2.5% = 0.21 ± 0.75; F(1,44) < 1), while for CD patients, the difference between detection and categorization was larger at 2.5% than at 10% contrast (10% = 0.38 ± 1.10; vs. 2.5% = 1.29 ± 0.75; F(1,44) = 10.76, *p* < 05.1). Then, we examined the three-way interaction considering only controls and NoCD patients. Once again, this interaction was not significant (F(1,44) < 1; NoCD patients: 10% = 0.10 ± 0.17; vs. 2.5% = 0.24 ± 0.81).

## Discussion

This study was aimed at investigating, in the central visual field, visual abilities in the detection and categorization of photographs of scenes and faces of patients suffering from POAG. The Results did not show the same outcomes for patients, depending on the presence of a central visual defect in static perimetry evaluation using Humphrey 24–2 SITA-Standard test. In comparison with age-matched controls, CD patients showed a deficit in both detection and categorization of low-contrast scene and face stimuli. Furthermore, the difference in performance between detection and categorization was greater for CD patients than for the controls, suggesting that the cost of high-level visual processes for patients was higher. It should be noted that, for the face categorization task, the results of CD patients for face stimuli at 2.5% contrast support those from other behavioral studies [6,11]. These studies showed that patients who were at an advanced stage of the disease exhibit a deficit in a face recognition task, but that patients could adapt to this deficit using specific eye movements patterns. However, in the present study, stimuli were presented for 100 ms, thus making it impossible to rely on eye movements strategies. Unexpectedly, for the categorization of face stimuli at 10% contrast, we did not observe significant difference between controls and CD patients. This result can be explained by the fact that the retinal sensitivity defect in CD patients does not cover the entire central 6° region, in which face stimuli were presented. Several studies on face perception have shown that face processing can be based on featural processing [21,22,23,24]. Thus, CD patients, even with their partial representation of the image, may rely on face features to perform a supraordinate categorization of human faces among other stimuli (animals and vehicles images). The partial, and not uniform, perimetry defect in the central visual field of CD patients could thus be strongly detrimental to the global perception of a stimulus displayed in the whole central visual field. Therefore, the study showed that CD patients have a deficit in categorization that surpasses their compromised detection. This suggests that visual tests based on stimuli detection may be not adequate to estimate the extent of visual disabilities of patients in their daily life. NoCD patients showed similar performance to age-matched controls for the detection and categorization of faces. For scenes, patients’ performances did not significantly differ from those of controls for the detection task, but they demonstrated a deficient categorization task. These results point to a high-level visual deficit that is not predicted by simple retinal sensitivity in the central visual field of patients. Patients participated in this experiment following their usual ophthalmological examination in which a central visual field defect was identified using a 24–2 SITA-Standard procedure of the Humphrey visual field analyzer. In clinical practice, if this procedure does not show a central visual field defect, a complementary 10–2 SITA-procedure is not performed. The latter could have resulted in a better detection of an early central defect [25].

A major conclusion of our results is that they suggest that a peripheral vision loss may induce a subtle high-level visual deficit in central vision. In spite of its low resolution, peripheral vision in normally sighted people is quite efficient and allows the recognition of scenes and objects, even at very high eccentricities (above 70°) [26, 27]. Furthermore, peripheral information could influence the recognition of information by central vision. Indeed, Boucart et al. [26] showed that the categorization of an object as an animal by central vision is facilitated when the animal is congruent to its scene background (relative to an incongruent scene background) in normally sighted aged people, but also in patients with aged-related macular degeneration (i.e. a disease characterized by loss of central vision).

The discrepancy in findings between the Scene and Face experiments for NoCD patients can be explained by many factors. First, the Scene experiment requires a subordinate categorization (indoors vs. outdoor scenes), while the Face experiment needs a categorization at the basic level (human faces vs. animals vs. vehicles). According to the cognitive economy principle, the basic level would be favored because it maximizes both similarity within a category (e.g. human faces are structurally very similar), and distinction between categories (e.g. human faces, vehicles and animals are structurally different). Thus, basic-level categorization would be the earliest and the more efficient categorization. A subordinate level categorization would be more costly, involving more cognitive and visual processes [28]. In our experiment, indoor and outdoor scenes have common physical characteristics, in particular similar visual cluttering and distribution of energy in terms of spatial frequency and dominant orientations. Their discrimination may thus rely on an additional process such as identification of details. An alternative explanation would be linked to real-life characteristics of these two categories. Behavioral and neuroimaging studies have shown that the periphery is more useful than central vision for categorizing natural scenes [29,30,31,32,33]. For example, young healthy subjects can categorize a scene as a beach, a forest or a street more accurately when only peripheral information is available (i.e. when the central portion of the scene is hidden by a circular scotoma) than when only the central portion of the scene is available [30]. In natural conditions, global scene perception is constant, encompassing the whole visual field, even when central vision is dedicated to a fine, object-directed visual task. Scenes can thus be considered as a category mostly calling upon peripheral vision, which is also suggested by an fMRI study [31] showing an overlap between regions activated by images of buildings and the cortical representation of the peripheral visual field. In contrast, the same study showed that faces preferentially activated the cortical representation of the central visual field. This may explain why NoCD patients were mostly impaired in the scene experiment, while face categorization performance was relatively highly preserved. It should be noted that our results for the face experiment support those from another behavioral study [6] showing that only POAG patients who were at an advanced stage of the disease exhibit a deficit in a face recognition task.

Based on the results of Lenoble et al. [7], we predicted that the deficit in categorization relative to detection would be stronger with a lower contrast level of stimuli. This hypothesis is also driven by the fact that patients often complain about extreme lighting conditions, like a darkened room, twilight, full sunlight or excessive artificial lighting [4,34]. From a neurophysiological point of view, parasol ganglion cells have higher contrast gain than midget ganglion cells. They are thus more sensitive to low luminance contrasts. These cells form the magnocellular pathway and are found mainly in the peripheral retina [35]. Glaucoma affects the parasol ganglion cells early in the disease. It could thus affect the magnocellular pathway and consequently cause a higher recognition deficit with low contrasts. However, results of the Scene experiment did not show a contrast effect. For NoCD patients, the difference between detection and categorization increased with a lower contrast (d’ difference increased from 0.96 for 10% to 1.92 for 2.5%). However, this was also the case for the controls (d’ difference increased from 0.57 for 10% to 1.29 for 2.5%). Although the effect of contrast was greater for patients, the statistical analysis did not show a significant interaction. For CD patients, the difference between detection and categorization was not statistically different between 10% and 2.5% and sensitivity showed a floor effect at 2.5%. Therefore, our study revealed a contrast effect on high-level processes involved in scene recognition in both aged controls and NoCD patients. Nevertheless it did not detect a greater contrast effect in NoCD patients than in the controls. It should be noted that the contrast levels used by Lenoble et al. [7] were 50% and 100%, i.e. higher and more distinctive than those used in the present study (2.5% and 10%). These very low contrast levels were initially chosen to simulate twilight conditions. They may have been too low. Hence they could have significantly biased effects. Further studies need to increase the number of contrast levels to establish the critical contrast range for which visual recognition difficulties in POAG patients are most likely to be measured.

Importantly, results of the present study showed that compared to age-matched controls, NoCD patients, demonstrated a deficit for scene categorization in their central vision. The detection of scenes was well-maintained. Thus, the deficit for categorization in central vision can hardly be explained by the sole retinal sensitivity loss for these patients. An alternative explanation might be that the progressive destruction of retinal ganglion cells in glaucoma can trigger trans-synaptic degeneration in the lateral geniculate nucleus and visual cortex [36,37,38]. This would cause structural and functional changes in high-level cerebral areas, affecting visual function as a whole. Increasing evidence from MRI studies supports the hypothesis of important anatomical and functional cortical changes linked to a neuronal degeneration in glaucoma [39,40,41,42,43,44,45]. For example, a voxel-based morphometry study [39] revealed that grey matter density of patients with POAG was reduced in the medial part of the anterior occipital cortex compared to controls. This corresponds to the approximate visual field defect projections of patients in the visual cortex. Studies using functional MRI retinotopic mapping allowed exploration of activation of the primary visual cortex, with respect to the representation of the visual field in patients with POAG. They revealed an alteration of the MRI signal consistent with the peripheral visual field loss as stated by perimetry [40]. In addition to cortical differences directly linked to retinal loss, other studies found abnormalities in many other visual and non-visual brain areas of POAG patients [41,44,45,46,47,34,35,36,37]. For example, a high resolution MRI study [44] showed that the optic nerve and the optic chiasm were thinner in patients than in controls. This study also showed that the grey matter density was reduced in the calcarine and lingual gyri. It was also decreased or increased in numerous temporal, frontal, and parietal cortical regions. Similarly, another MRI study [41] showed a grey matter atrophy in cortical regions involved in object and scene recognition (i.e. the lateral occipital complex or LOC [48] and the parahippocampal place area or PPA, respectively [17,49,50]). The study revealed a decrease in functional connectivity within the visual cortical network but also within the working memory and the attention networks. Concerning specifically the visual cortical network, a resting state fMRI study [47] reported alteration of functional connectivity between early visual areas and high-order visual areas dedicated to visual recognition like the fusiform and the inferior temporal gyri. Overall, MRI studies of glaucoma point out evidence for a large range of structural and functional modifications in both the visual and non-visual brain. In particular, alterations in integrative visual areas supporting the final step of visual recognition in normal brain could explain why patients with or without central visual defects may suffer from scene recognition impairment. Therefore, studying functional and structural abnormalities of integrative high-level visual areas (e.g. the scene selective PPA) in relation to behavioral measures seems to be an interesting approach for the understanding of visual scene recognition deficits in POAG patients.

In conclusion, this study highlights an alteration of visual recognition in the central visual field of patients with glaucoma, despite the absence of a central defect in static perimetry evaluation using Humphrey 24–2 SITA-Standard test. Our experimental paradigm compares the abilities of POAG patients to detect and categorize the same stimuli, allowing support of the hypothesis of a deficit in categorization even when detection abilities are intact.
